# Highly efficacious antiviral protection of plants by small interfering RNAs identified *in vitro*

**DOI:** 10.1093/nar/gkz678

**Published:** 2019-08-21

**Authors:** Selma Gago-Zachert, Jana Schuck, Claus Weinholdt, Marie Knoblich, Vitantonio Pantaleo, Ivo Grosse, Torsten Gursinsky, Sven-Erik Behrens

**Affiliations:** 1 Institute of Biochemistry and Biotechnology, Martin Luther University Halle-Wittenberg, Halle/Saale D-06120, Germany; 2 Department of Molecular Signal Processing, Leibniz Institute of Plant Biochemistry, Halle/Saale D-06120, Germany; 3 Institute of Computer Science, Martin Luther University Halle-Wittenberg, Halle/Saale D-06120, Germany; 4 Institute for Sustainable Plant Protection-Consiglio Nazionale delle Ricerche, Research Unit of Bari, Bari I-70126, Italy; 5 German Centre for Integrative Biodiversity Research (iDiv) Halle-Jena-Leipzig, Leipzig D-04103, Germany

## Abstract

In response to a viral infection, the plant’s RNA silencing machinery processes viral RNAs into a huge number of small interfering RNAs (siRNAs). However, a very few of these siRNAs actually interfere with viral replication. A reliable approach to identify these immunologically effective siRNAs (*esiRNAs*) and to define the characteristics underlying their activity has not been available so far. Here, we develop a novel screening approach that enables a rapid functional identification of antiviral *esiRNAs*. Tests on the efficacy of such identified *esiRNAs* of a model virus achieved a virtual full protection of plants against a massive subsequent infection in transient applications. We find that the functionality of *esiRNAs* depends crucially on two properties: the binding affinity to Argonaute proteins and the ability to access the target RNA. The ability to rapidly identify functional *esiRNAs* could be of great benefit for all RNA silencing-based plant protection measures against viruses and other pathogens.

## INTRODUCTION

Virus-induced diseases cause significant reductions in both crop quality and yield worldwide (1,2). The global trade and climate change exacerbate this situation by supporting the spread of vectors and pathogens into new areas (3).

A major component of the plant’s immune response against viral infections is the RNA silencing process (4,5). Structured regions of viral mRNAs and genomes and/or double-stranded (ds) RNA molecules induce RNA silencing in plants (6–9). DsRNAs are produced, for example, during infections with (+)-strand RNA viruses, which represent the vast majority of plant-infecting viruses (10). Genome replication of these viruses occurs in the cell’s cytoplasm and involves a two-step process via (−)-strand RNA and dsRNA replication intermediates. Cellular Dicer-like ribonucleases such as DCL4 and DCL2 can detect and process dsRNAs into virus-derived small interfering duplex RNAs (*vsiRNAs*) of 21 and 22 nt, respectively (11,12). Associated with the removal of the vsiRNA’s passenger strand, the remaining guide strand is incorporated into Argonaute (AGO) endonucleases (13), the core components of RNA-induced silencing complexes (RISC).

The siRNA guide strand directs the RISC to complementary sequences in the cognate viral RNA enabling AGO-catalyzed cleavage (‘slicing’) (14–16). RISC-mediated slicing of viral RNAs then further amplifies the silencing response via the production of secondary siRNAs involving host-encoded RNA-dependent RNA polymerases and DCLs (17–20). Of the AGO proteins identified in plants, AGO1 and AGO2 have been found to be important antiviral effectors against plant viruses possessing an RNA genome (21).

The successful induction of antiviral RNA silencing crucially depends on the capability of the vsiRNA molecules to interfere specifically and effectively with viral protein translation and/or RNA replication. Previous studies suggested that from the huge pool of vsiRNAs generated during plant infection and DCL-mediated processing of dsRNAs only a very small subset, hereinafter referred to as ‘effective siRNAs’, *esiRNAs*, act antivirally and support RNA slicing (9,16,22,23). However, there has been no simple and systematic approach to distinguish the immunologically active *esiRNAs* from the bulk of other vsiRNAs.

Here, we present a new *in vitro*-based procedure that enables a rapid and reliable identification as well as a conclusive functional characterization of *esiRNAs* of a specific pathogen. We find that two main properties determine the efficiency of *esiRNAs* and demonstrate that the identified *esiRNAs* generate excellent antiviral protection rates in the plant.

A systematic identification and application of *esiRNAs* might significantly improve plant protection measures based on RNA silencing. Specifically, the use of *esiRNAs* might further enhance the potential of topical versus transgenic applications and reduce the risk of pathogen resistance breakage.

## MATERIALS AND METHODS

### Cell culture and preparation of BYL

*Nicotiana tabacum* BY2 cells were cultured at 23°C in Murashige-Skoog liquid medium (Duchefa, Haarlem, The Netherlands). Cytoplasmic extract (BYL) was prepared from the evacuolated cells as described (24,25).

### Plasmid constructs

To generate a cDNA clone encoding TBSV (−)-strand, the complete TBSV (T100) cDNA was PCR-amplified (plasmid A, kindly provided by Herman B. Scholthof, Texas A&M University) with a reverse primer containing an upstream T3 promoter sequence and a forward primer that introduced an additional XbaI site adjacent to the TBSV sequence. By ApaI/SmaI cloning, the original TBSV cDNA sequence including the T7 promoter was removed from plasmid A and replaced by the PCR fragment generating plasmid B. To generate GFP mRNA fragments containing complementary sequences of TBSV siRNAs, we first replaced the target site of gf698 siRNA in plasmid pGFP-C1 by two BpiI sites. This was done by inserting two PCR fragments between Eco72I and BamHI. The cDNA fragments (double-stranded oligonucleotides) encoding the respective TBSV target site sequences were then inserted into the BpiI-digested plasmid. To generate a binary vector for the expression of TBSV genomic RNA in plants, we first cloned a sequence encoding the *Hepatitis delta virus* (HDV) ribozyme right downstream of the TBSV cDNA. This was done by PCR amplification of the ribozyme-encoding cDNA from plasmid pWNVRepliconHDVr (26), followed by inserting the fragment in SmaI-linearized plasmid A (see above). Subsequently, the complete TBSV cDNA including the ribozyme-encoding sequence was PCR-amplified and inserted together with a *Cauliflower mosaic virus* (CaMV) 35S promoter module into the BsaI-cut binary vector pVM-BGW (27).

### *In vitro* transcription

*AGO* mRNAs were synthesized in the presence of monomethylated cap analog m^7^GP_3_G (Jena Biosciences, Jena, Germany) from SwaI-linearized plasmid constructs (23,28) using SP6 RNA polymerase (Thermo Fisher Scientific, Waltham, MA). Transcripts encoding the firefly luciferase mRNA were generated by SP6 RNA polymerase from the XhoI-linearized plasmid pSP-luc(+) (Promega, Madison, WI). Transcription reactions and subsequent treatment of the transcripts were performed by using standard procedures. TBSV genomic RNA (T100) was synthesized by T7 RNA polymerase from SmaI-linearized plasmid A (25). TBSV (-)RNA was synthesized by T3 RNA polymerase from XbaI-linearized plasmid B (see above). Sense and antisense TBSV RNA fragments were produced by T7 RNA polymerase from PCR products where the T7 promoter sequence was included in the forward or reverse PCR primers (see Supplementary Table S1 for oligonucleotide sequences). Labeling of RNAs was performed by *in vitro* transcription in the presence of 0.5 μCi/μl [α-^32^P]-CTP (3000 Ci/mmol). dsRNAs were produced by mixing equimolar amounts of sense and antisense transcripts in STE buffer (10 mM Tris/HCl, pH 8.0, 100 mM NaCl, 1 mM EDTA), heating for 2 min at 94°C and decreasing the temperature to 25°C within 30 min. The ds nature of the annealed RNAs was confirmed by treatment with 0.05 U/μl RNase T1 (Thermo Fisher Scientific) for 15–60 min at 37°C and subsequent gel analysis.

### DCL assay

To generate TBSV siRNAs *in vitro*, 1.25 μg ds genomic TBSV RNA was incubated for 2 h at 25°C in a 50 μl reaction containing 50% (v/v) BYL, using conditions previously described (23,28). Total RNA was isolated from the reaction by treatment with 20 μg proteinase K in the presence of 0.5% SDS for 30 min at 37°C, followed by phenol/chloroform extraction and ethanol precipitation. NGS was performed with an Illumina Highscan at the Core Unit DNA-technologies of the University of Leipzig, Germany (Dr Krohn). Data were obtained from three independent experiments.

### Isolation of AGO-bound vsiRNAs

To generate siRNA-programmed AGO/RISC *in vitro*, 5 pmol *Nicotiana tabacum AGO1* or *Arabidopsis thaliana AGO2* mRNA was translated in the presence of 5 μg TBSV dsRNA in a 200 μl reaction containing 50% (v/v) BYL under the above-described conditions. Samples were mixed with an equal volume of immunoprecipitation buffer (IPB, 20 mM Hepes/KOH, pH 7.6, 150 mM NaCl, 0.5% (v/v) NP-40, 1 mM DTT) and 20 μl anti-FLAG M2 affinity gel (Sigma-Aldrich, St. Louis, MO). Following overnight incubation at 4°C with gentle agitation, the resin was washed four times with IPB and once with IPB containing 300 mM NaCl. RNA was isolated and analyzed as described above for the DCL assay. Data were obtained from two different AGO1 and three different AGO2 experiments.

### Bioinformatics analyses of NGS data

Raw reads were adaptor clipped using cutadapt (v1.12) (29) and quality filtered (-q 20) and trimmed (-l 15) using sickle (https://github.com/najoshi/sickle). The remaining and truncated reads were then mapped onto a combined reference of the tobacco genome (Ntab-BX_AWOK-SS.fa) and the TBSV sequence (TBSV.fa) using the bowtie aligner (v1.1.2) (30) allowing at most three mismatches (-n 3) and reporting only alignments in the best alignment ‘stratum’ (–best –strata). Mappings onto the TBSV sequence were extracted using sambamba (v0.6.3) (31) (-view -f TBSV), and only these mappings were used for further analyses using the software environment R (http://www.R-project.org/) and the R package Rsamtools (http://bioconductor.org/packages/release/bioc/html/Rsamtools.html). The set of mapped reads were partitioned into six sets depending on the read-lengths (21, 22 or 24 nucleotides) and the mapped strand (positive or negative), and the starting positions of the mapped reads were counted as peak expression. Reads were stored in 5′ to 3′ orientation in the Sequence Alignment Map (SAM) format, so the first (last) nucleotide was counted for reads mapped onto the positive (negative) strand. All statistical analyses were performed using custom R scripts executed in R Studio Server. Each of these error bars was calculated as standard error of the mean applying the *std.error* function of the *plotrix* (32) package.

### Production of siRNAs

The control siRNA gf698 siRNA targeting the GFP mRNA was described earlier (33). RNA oligonucleotides (Supplementary Table S2) to generate the here examined siRNAs were purchased from Biomers (Ulm, Germany). To produce siRNA duplexes, guide and passenger strands incubated for 1 min at 90°C and annealed for 60 min at 37°C in annealing buffer (30 mM Hepes/KOH, pH 7.4, 100 mM KOAc, 2 mM MgOAc).

### *In vitro* slicer assay

AGO/RISC programmed with a specific siRNA were generated as described above in a 20 μl reaction containing 50% (v/v) BYL, 0.5 pmol *N. benthamiana AGO1L* or *AGO2* mRNA (28,34) and 100 nM siRNA duplex. After 2.5 h at 25°C, 2 μg of firefly luciferase (competitor) mRNA and the ^32^P-labeled target RNA (20 fmol) were added, and the cleavage reaction performed for 15 min. Total RNA was isolated by treating the reaction with 20 μg proteinase K in the presence of 0.5% SDS for 30 min at 37°C, followed by chloroform extraction and ethanol precipitation. RNAs from assays with full-length TBSV RNA as target were separated on 1.5% denaturing agarose gels; all other RNA samples were separated on 5% TBE polyacrylamide gels containing 8 M urea. ^32^P-labeled target RNAs and cleavage products were visualized by phosphor-imaging (Storm 860, Molecular Dynamics).

### Plant vaccination and TBSV challenge

To express selected siRNAs as artificial microRNAs in plants, the corresponding sequences were introduced into the binary vector pMDC32B-AtMIR390a-B/c (35) and the resulting constructs transformed into *A. tumefaciens* strain GV3101. Individual colonies were grown overnight in selective medium and subsequently used to inoculate 30-ml induction medium (LB supplemented with 10 mM MES, pH 5.6, 20 μM acetosyringone and antibiotics). Cultures were grown overnight at 30°C until they reached an OD_600_ of 0.5–0.7, harvested by centrifugation for 10 min at 1780 *g* and re-suspended in an appropriate volume of infiltration medium (LB supplemented with 10 mM MES, pH 5.6, 10 mM MgCl_2_, 150 μM acetosyringone) to obtain an OD_600_ of 1.0. Suspensions were incubated at room temperature for at least 3 h and 2 ml were infiltrated into two leaves of 4- to 6-week-old *N. benthamiana* plants using a 1 ml needleless syringe. Forty-eight hours later, each of the two leaves was infiltrated with two times 50 μl *A. tumefaciens* carrying a binary vector containing the full-length TBSV (T100) cDNA downstream of a CaMV 35S promoter, followed by a *Hepatitis delta virus* ribozyme and a 35S terminator sequence. Bacterial suspensions were prepared as described above; shortly before infiltration, they were 1:1000 diluted with cultures containing an mCherry ORF instead of TBSV cDNA. After infiltration, plants were grown in a chamber (CLF Plant Climatics, Wertingen, Germany) for 14 h at 23°C, 90–100 μmolm^−2^s^−1^ light (at shelf level) and for 10 h at 21°C in the dark. Plants were daily monitored for symptom development for 5 weeks (25 days in case of the RDR6i plants). Mechanical inoculation of TBSV to *N. benthamiana* plants was performed by using infectious *in vitro* transcripts. Prior to the application of the viral RNA, the leaves were dusted with carborundum powder. TBSV RNA (2 ng/μl) was mixed with an equal volume of inoculation buffer (30 mM K_2_HPO_4_, pH 9.2, 50 mM glycine) and 2.5 μl two times rubbed onto the surface of the previously infiltrated leaves. The treated leaves were rinsed with water and the plants grown and monitored as described above.

## RESULTS

For the identification and functional characterization of *esiRNAs*, we used an *in vitro* system of cytoplasmic extracts from *N. tabacum* BY-2 protoplasts (24). The so-called BY-2 lysate (BYL) recapitulates the primary RNA silencing pathway in the following manner. DsRNAs are processed by extract-endogenous DCLs (23,36) when added to the lysate, and active RISC can be assembled with an *in vitro*-translated AGO protein of choice (23,33). Following the programming with small RNAs (e.g. siRNAs or miRNAs), which may be either endogenously DCL-generated as described above or exogenously added as synthetic molecules, the functionality of *in vitro*-generated RISC can be tested in a ‘slicer assay’ with a chosen target RNA (23,28,37) (examples can be found below). We chose *Tomato bushy stunt virus* (TBSV) (38) as a model pathogen, a (+)-strand RNA Tombusvirus showing a broad tropism and distinctive pathogenesis.

Considering its capability of acting as a silencing inducer and its homogeneous composition consisting of equal quantities of (+)- and (−)-strand RNA, we performed all of the experiments described below with TBSV dsRNA molecules. We generated the dsRNAs by *in vitro* transcription and annealing of RNA molecules corresponding to the full-length (+)-strand TBSV genome (4778 nt) and the complementary (−)-strand replication intermediate (Materials and Methods).

### DCL-dependent generation of vsiRNAs (‘Dicer Assay’)

**Figure 1.**
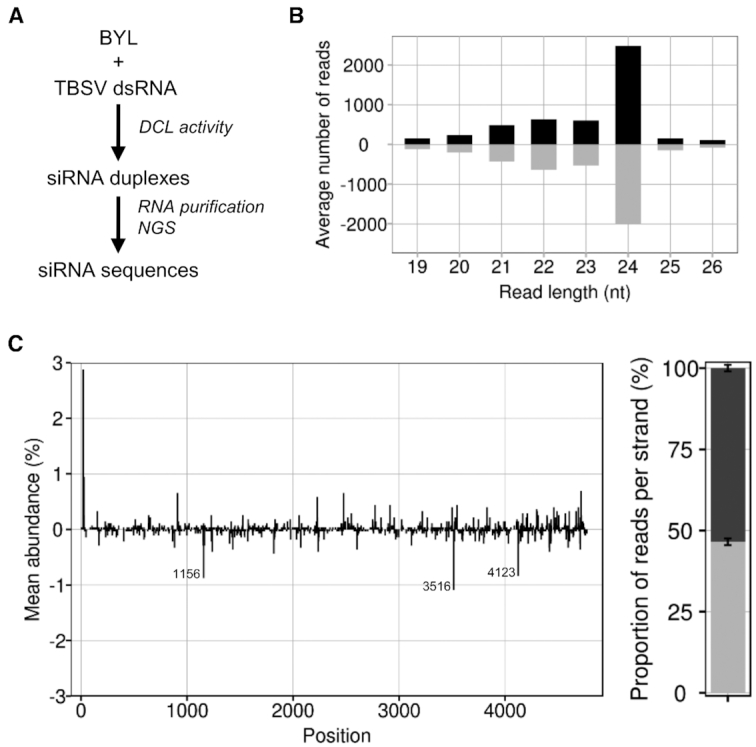
DCL-mediated generation of TBSV siRNAs in BYL. (**A**) Schematic representation of the *in vitro* ‘Dicer assay’ performed with double-stranded TBSV RNA. (**B**) Size distribution of sequenced TBSV siRNAs. Bars above the axis represent siRNAs derived from viral (+)RNA, bars below the axis represent siRNAs derived from viral (-)RNA. (**C**) Distribution and abundance of the sequenced 21 nt vsiRNAs aligned to the TBSV genome. Peaks above the axis represent siRNAs derived from viral (+)RNA, peaks below the axis represent siRNAs derived from viral (-)RNA. The peaks indicate the position of either the 5′ nucleotide of a vsiRNA with respect to the TBSV genome (in case of (+)vsiRNAs) or the TBSV genome position complementary to the 5′ nucleotide of a vsiRNA (in case of (-)vsiRNAs). The locations of the three most abundant (-)vsiRNA reads are specified. The chart on the right represents the relative abundance of the sum of (+) and (-)RNA derived siRNAs, respectively. Data represent mean ± S.E.M.

In the first approach, we exposed the TBSV dsRNA to the BYL to generate all types of vsiRNAs via the extract-endogenous DCLs. For reasons explained below, we chose conditions that enable the *in vitro* translation of proteins in the extract (25). Subsequently, we extracted the total RNA and identified DCL-generated vsiRNAs by next-generation sequencing (NGS) (Materials and Methods; Figure 1A). The assay generated vsiRNAs with sizes of 21, 22 and 24 nt of which the 24 nt siRNAs were the predominant species (Figure 1B). These results confirmed that DCL3 (generating 24 nt siRNAs) as well as DCL4 and 2 are all active in the BYL (36).

In the subsequent analyses, we focused on the 21 nt siRNAs as the most important antiviral siRNA species (8,39,40). In Figure 1 and below, the generated vsiRNAs were named by the position of their 5′ end aligned to the TBSV genome. We found from the NGS data that vsiRNAs derived from the (+)-strand of the dsRNA, referred to as (+)vsiRNAs, and vsiRNAs derived from the (−)-strand of the dsRNA, referred to as (-)vsiRNAs, were produced in an almost balanced ratio. In addition, we identified a small number of moderately preferred DCL4 cleavage sites suggesting some sequence preferences of DCL4 within the TBSV dsRNA (Figure 1C). Indeed, the generated (-)vsiRNAs showed a slightly elevated GC content: for example, with the 50 most abundant vsiRNAs, the average GC content was 50.4% compared to 48.2% in the TBSV genome. In agreement with earlier findings of Ho *et al.* (41) with *Turnip mosaic virus* (TuMV), these data support the idea that GC-rich sequences are moderately preferred DCL target sites.

### Identification of RISC-incorporated vsiRNAs (‘RISC-IP’)

Previous reports suggested that *in vitro* assembled AGO/RISC can be isolated from BYL (33). In view of these findings, we conducted pilot studies, in which we generated a FLAG-tagged version of AGO1 by *in vitro* translation of the corresponding mRNA in the lysate; the translation was carried out in the presence of a synthetic siRNA, gf698, which targets the mRNA of green fluorescent protein (GFP). Next, we isolated the generated siRNA-associating (‘programmed’) AGO1/RISC via immunoprecipitation using an anti-FLAG affinity gel and demonstrated it to be functional in a slicing assay with the GFP mRNA (Supplementary Figure S1).

**Figure 2.**
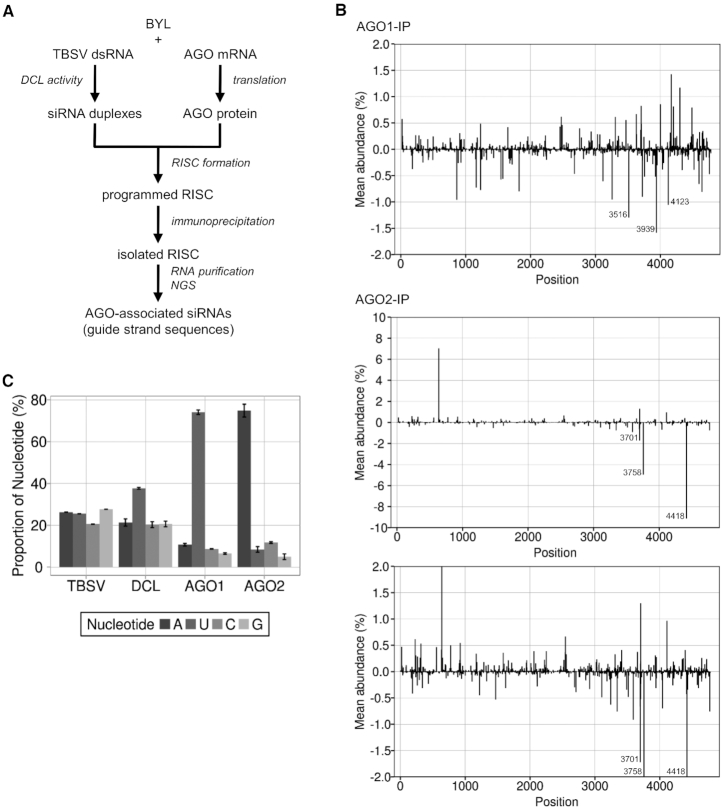
Identification of RISC-incorporated TBSV siRNAs. (**A**) Schematic representation of the RISC immunoprecipitation procedure. FLAG-tagged AGO1 or AGO2 was generated by *in vitro* translation in the BYL in the presence of ds TBSV genomic RNA. The dsRNA was processed into vsiRNAs by the extract-endogenous DCLs and RISCs formed (‘programmed’) with these vsiRNAs. The RISCs were immunoprecipitated using an immobilized anti-FLAG antibody. Subsequently, siRNA guide strands were isolated and analyzed by NGS. (**B**) Distribution of sequenced 21 nt siRNA guide strands from immunoprecipitated AGO1/RISC or AGO2/RISC aligned to the TBSV genome. For better comparability, the additional image below shows a section of the AGO2 data with the same scaling as the AGO1 data. Peaks above the axis represent siRNAs derived from viral (+)RNA, peaks below the axis represent siRNAs derived from viral (-)RNA (see Figure 1 for the assignment of the peaks). The three most abundant (-)vsiRNA reads are shown. (**C**) Relative frequency of the respective 5′ terminal nucleotides of AGO1- and AGO2-associated 21 nt TBSV siRNA guide strands. The abundance was compared to the nucleotide composition of TBSV dsRNA and to the relative frequency of the 5′ terminal nucleotides of all 21 nt TBSV siRNAs that were generated by BYL-endogenous DCLs (see Figure 1). Data represent mean ± S.E.M.

In the following steps, we focused on the identification of AGO/RISC-associated TBSV vsiRNAs. For this purpose, we processed TBSV dsRNA again by the BYL-endogenous DCLs to produce the complete spectrum of vsiRNAs (Figure 1). Simultaneously, we generated FLAG-tagged AGO1 or AGO2 proteins *in vitro* as described above, immunoprecipitated the ‘siRNA programmed’ AGO/RISC and sequenced the bound vsiRNAs by NGS (Figure 2A).

It has been shown that, directed by the RNA’s size and the 5′ terminal nucleotide, AGO1 favorably binds 21 nt siRNAs with a 5′ uridine, while AGO2 preferentially binds 21 nt siRNAs with a 5′ adenosine (42,43). We observed here that vsiRNAs with a 5′U were indeed preferentially enriched in the AGO1/RISC, whereas the AGO2/RISC contained primarily vsiRNAs with a 5′A (Figure 2C and Table 1). When comparing the NGS data of the ‘DCL assay’, which recorded the total number of DCL-generated vsiRNAs (Figure 1C), to the NGS data of these ‘RISC-IP’, we found that numerous vsiRNAs were significantly enriched in the RISC, indicating the highly preferential binding of these vsiRNAs to AGO1 or AGO2 (Figure 2B).

**Table 1.** Most abundant AGO/RISC-associated TBSV (-)siRNAs; datasets categorized as explained in the text and below

Dataset 1^a^					
AGO1			AGO2		
vsiRNA	5′ nt	Mean abundance (%)	vsiRNA	5′ nt	Mean abundance (%)
3939	U	1.59	4418	A	9.13
3516	A	1.30	3758	A	4.96
4123	U	1.06	3701	A	1.72
864	U	0.96	3593	A	0.92
3257	U	0.95	3342	A	0.76
3722	U	0.90	4771	A	0.76
4643	U	0.81	4044	A	0.70
1823	U	0.80	3698	A	0.66
1230	U	0.77	3243	A	0.63
1164	U	0.73	1470	A	0.53
Dataset 2^b^					
AGO1			AGO2		
vsiRNA	5′ nt	log2 Fold change	vsiRNA	5′ nt	log2 Fold change
3722	U	7.72	3701	A	8.31
1575	U	7.04	4418	A	7.55
2678	U	6.77	3758	A	7.52
3939	U	6.54	3698	A	6.93
4336	U	6.54	3107	A	6.49
4037	U	6.11	1221	A	6.38
1680	U	5.98	4415	A	6.30
3927	U	5.92	186	A	6.27
343	U	5.87	4771	A	6.13
3257	U	5.80	3943	A	5.86
Dataset 3^c^					
AGO1			AGO2		
vsiRNA	5′ nt	Mean abundance (%)	vsiRNA	5′ nt	Mean abundance (%)
179	U	0.38	4044	A	0.70
1717	U	0.29	3243	A	0.63
4052	U	0.26	1470	A	0.53
885	U	0.26	3704	A	0.53
4667	U	0.21	2710	A	0.39
1324	U	0.20	3492	A	0.34
1252	U	0.19	2921	A	0.33
1574	U	0.19	340	A	0.31
4585	U	0.19	261	A	0.27
1432	U	0.17	432	A	0.23

^a^vsiRNAs were listed exclusively according to their frequency in AGO/RISC immunoprecipitation.

^b^vsiRNAs were sorted by their accumulation in AGO/RISC immunoprecipitation compared to their frequency in the DCL assay.

^c^vsiRNAs, not detectable in the DCL assay but readily detectable in the AGO/RISC immunoprecipitation, were listed according to their frequency in the latter.

### Functional characterization of AGO-associated vsiRNAs

It has been observed that during TBSV replication only the (+)-strand viral RNA molecules are accessible to RISC-mediated degradation, whereas the (−)-strand intermediates are not (23). This observation was attributed to the inability of RISC to access the membrane-enclosed viral replication complexes (16,44–46). Accordingly, we carried out the following studies exclusively with (-)vsiRNAs, which are expected to target the viral (+)-strand RNAs.

In order to gain initial insights into the properties of NGS-associated (-)vsiRNAs, we first classified them according to the following criteria (Table 1). In a first dataset, we classified the vsiRNAs exclusively according to the frequency of their association with the AGO/RISC; for some of these vsiRNAs, it could be assumed that they only bind well to the AGOs because they were efficiently processed by the DCLs from the dsRNAs and were present in correspondingly high quantities during RISC formation (Figure 1). In a second dataset, we classified the vsiRNAs by their accumulation in AGO/RISC immunoprecipitation compared to their frequency in the DCL assay; for vsiRNAs that were detected here in significantly higher amounts in AGO/RISC, a high affinity to the respective AGO proteins could be assumed. In a third dataset, we classified all vsiRNAs that were not detectable in the DCL assay but were readily detectable in the AGO/RISC-IP. With these vsiRNAs, a robust enrichment in AGO/RISC indicated an even higher AGO affinity than with the vsiRNAs of the second dataset. As expected, we observed overlaps between datasets 1 and 2, as well as between 1 and 3.

**Figure 3.**
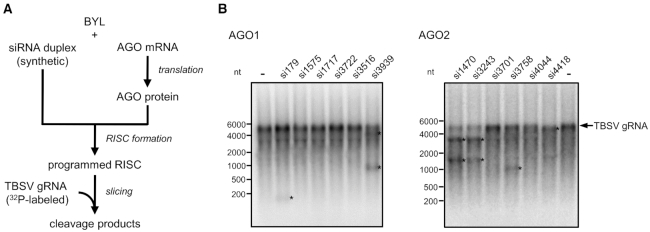
vsiRNAs with high affinity to AGO mediate *in vitro* cleavage of TBSV genomic RNA with different efficiency. (**A**) Schematic representation of the ‘slicer assay’. AGO1 or AGO2 was generated by *in vitro* translation in the BYL in the presence of synthetic siRNA duplexes generating RISCs that were programmed with these siRNAs. Subsequently, ^32^P-labeled, full-length TBSV RNA was added and analyzed for siRNA-mediated cleavage by denaturing PAGE and autoradiography. (**B**) Results of a representative ‘slicer assay’ performed with different TBSV siRNAs that were found to be abundant in AGO/RISC immunoprecipitations (see also Table 1 and Figure 2). A cleavage event was indicated by (i) an apparent reduction in the amount of target viral RNA and/or (ii) the occurrence of one or two cleavage product(s) having the expected size(s) (indicated by asterisks).

Considering each of these vsiRNA-categories as potential sources of *esiRNAs*, we tested the two or three top candidates of each dataset (Table 1) for activity in slicer assays with the full-length TBSV genome (Figure 3A; ‘Materials and Methods’ section). To this end, we probed synthetic versions of vsiRNAs 3939, 3722, 3516, 1575, 179 and 1717 (all except for vsiRNA3516 having a 5′U), which were identified in the immunoprecipitated AGO1/RISC, with AGO1 for slicer activity. Likewise, we probed synthetic versions of vsiRNAs 4418, 3758, 3701, 4044, 3243 and 1470 (all having a 5′A), which were identified in the immunoprecipitated AGO2/RISC, with AGO2 for slicer activity. We found that readily detectable cleavages of the viral RNA were measured with vsiRNAs 179 and 3939 (AGO1) as well as with vsiRNAs 3243, 4418, 1470 and 3758 (AGO2). The remaining vsiRNAs showed no, or nearly no, activity (Figure 3B). Accordingly, from each of the three categories of AGO1- or AGO2-enriched (-)vsiRNAs, some were active, while others were not. These observations suggested that the affinity of a particular vsiRNA to an AGO protein is an important criterion that determines its activity in RISC-mediated slicing. On the other hand, it became clear that the affinity of a siRNA to AGO is not the only activity criterion. A particular case was vsiRNA3516, which has a 5′A, but was unexpectedly selected with AGO1. VsiRNA3516 was not active with AGO1 in the *in vitro* slicer assay (Figure 3), but when tested with AGO2/RISC, it was functional (not shown; ‘Discussion’ section).

The fact that siRNAs that bind at high affinity to AGO may nevertheless remain slicing-inactive might be explained by the fact that these siRNAs were complementary to non-accessible RNA elements in the viral genome. To address this possibility, we incorporated the target sequences of each of the above-described vsiRNAs into the context of the GFP mRNA and repeated the slicer assay with these target-mRNAs. Interestingly, all of the vsiRNAs mediated slicing under these conditions, indicating that the previously observed inactivity of siRNAs on the TBSV genome could be explained by the inaccessibility of the viral RNA to RISC (Supplementary Figure S2).

**Figure 4.**
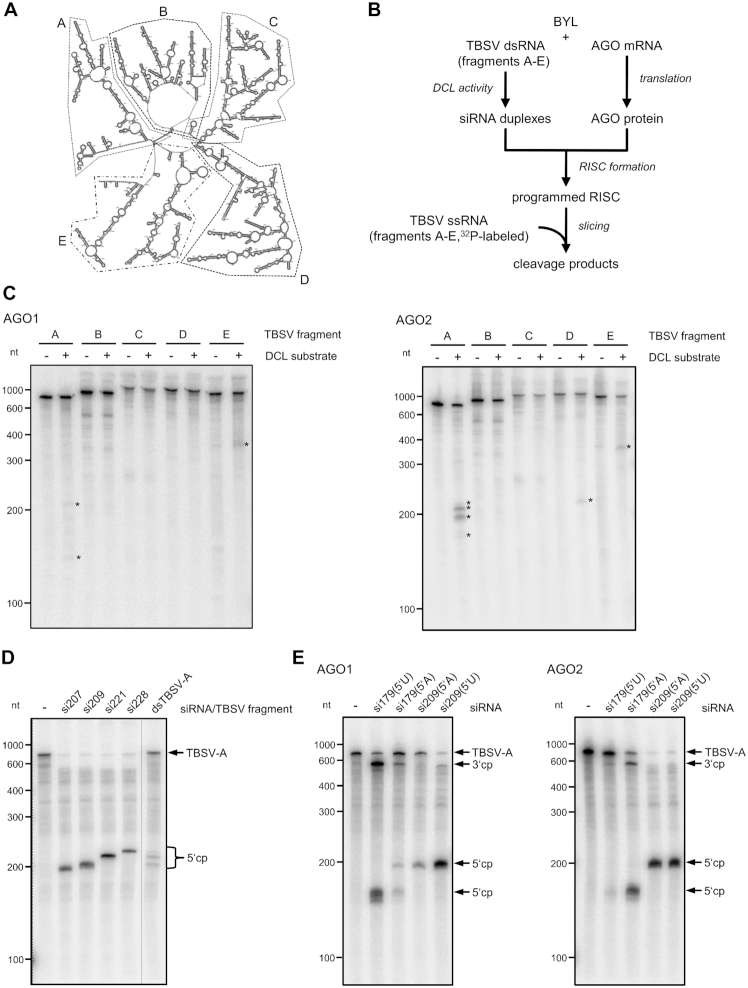
Identification of vsiRNAs targeting particularly susceptible sites in the TBSV genome. (**A**) Secondary structure model of a TBSV RNA genome (47). Fragments A–E that were used in the slicer assay described below are indicated by dashed or spotted lines. (**B**) Schematic representation of the ‘slicer assay’ using a BYL-generated pool of TBSV siRNAs. Here, the AGO1 or AGO2/RISC was programmed with vsiRNAs that were produced from one of the ds TBSV fragments A–E by the BYL endogenous DCLs. As a control, the RISC-programming reaction was carried out in the absence of TBSV fragments. Subsequently, the corresponding ^32^P-labeled, single-stranded fragment transcript was added to the reaction and analyzed for cleavage by denaturing PAGE and autoradiography. (**C**) Results of the ‘slicer assay’ applying the BYL-generated vsiRNA pools. Detectable cleavage products are indicated by asterisks. (**D**) ‘Slicer assay’ performed with synthetic siRNAs that were selected as candidates for efficient cleavage of TBSV RNA fragment A. The assay was carried out as described in Figure 3 with AGO2. To assign the cleavage products (indicated as cp), a slicer assay containing the pool of ds fragment A-generated vsiRNAs (see B) was performed in parallel. (**E**) ‘Slicer assay’ performed with AGO1 or AGO2 and synthetic siRNAs 179 and 209 carrying different 5′ nucleotides. The assay was carried out with TBSV fragment A as target RNA as described in Figure 3. Cleavage products (cp) are indicated.

These findings encouraged us to apply the *in vitro* system to (i) attempt to identify sites in the TBSV genome that are particularly susceptible to RISC and to (ii) attempt to identify those vsiRNAs that target these sites. Since *in vitro* slicing experiments with the full-length TBSV genome have been found to be too insensitive for the detection of cleavage products produced by individual species of a vsiRNA pool (23), we applied here five similarly sized fragments (A–E) of the genome considering the proposed global domain organization of the TBSV RNA (47) (Figure 4A).

We exposed dsRNA versions of A–E to the BYL DCLs and programmed *in vitro*-assembled AGO1/RISC or AGO2/RISC with the generated vsiRNA pools as described above (Figures 1 and 2). Next, we added the respective radiolabeled single-stranded transcripts as RISC target-substrates and analyzed for cleavage (Figure 4B). We found in the experiments performed with AGO1/RISC that cleavage products were detectable with some of the applied RNAs, but we also found that the amounts were low and at the detection limit of the assay. We obtained analogous results with AGO2/RISC and regions B–E. In contrast, in the assays with AGO2/RISC and region A including the ∼740 nt 5′ terminal region of the TBSV genome, we found a pattern of highly abundant cleavage fragments with sizes of ∼200 nt (Figure 4C).

This observation indicated that the 5′ portion of the TBSV genome contained easily accessible sites for AGO2/RISC; hence, we decided to pinpoint candidate siRNAs putatively involved in the generation of these fragments by reanalyzing the NGS data of the AGO2/RISC-IP. For this purpose, we considered that RISC-mediated cleavage of a target RNA takes place between positions 10 and 11 of the complementary siRNA guide strand (48,49) and found in this way candidate (-)vsiRNAs with a 5′A at positions 207, 209, 221 and 228. Interestingly, all of these vsiRNAs belonged to the dataset of category 3 (Table 1), i.e. all of these vsiRNAs were untraceable in the DCL-assay but detectable in the AGO2/RISC-IP (Supplementary Figure S3).

When we tested the synthetic siRNAs in a slicer assay with AGO2/RISC and the region A target-RNA, each of them showed a robust slicer-activity (Figure 4D). Interestingly, the sizes of the fragments generated by AGO2/RISC loaded with vsiRNA209 or vsiRNA221 closely corresponded to the sizes of the most dominant fragments generated in the corresponding slicing assay in which AGO2/RISC was programmed with the entire TBSV region A-derived vsiRNA pool (Figure 4D). When we repeated the assay with smaller inputs of siRNAs 207, 209, 221 and 228 and in the presence of competitor gf698 siRNA, we found that vsiRNA209 was most effective, supporting the observations of Figure 4D. We also found that vsiRNA209 exhibited high activity in the slicing assay using the full-length TBSV genome (Supplementary Figure S4).

These findings suggest that the applied screening approach enables the identification of several AGO1- and AGO2-selected *esiRNAs* from the whole pool of TBSV vsiRNAs that were highly active in *in vitro* slicer assays with the viral target RNA. We found that a high affinity to AGO proteins is necessary, but not sufficient, for the functionality of an *esiRNAs* and that an additional requirement is the accessibility of the target RNA for the siRNA guide strand-containing RISC. The BYL-based *in vitro* assay proved to be useful in detecting such RISC-accessible sites.

### Switching of the *esiRNA’s* AGO specificity

To scrutinize the observation that the accessibility of a target RNA to RISC affects the capability of an siRNA to function as an *esiRNA*, we next aimed to test if the sites within the viral genome that are accessible to AGO2/RISC with a specific siRNA were also accessible to AGO1/RISC containing the identical siRNA and *vice versa*.

We generated isoforms of the most effective AGO2-selected vsiRNA209 that contained a 5′U (instead of an A) in the guide strand, and a complementary A (instead of a U) in the passenger strand. Similarly, we modified the vsiRNA179 (AGO1-selected) by changing the 5′U of the guide strand to an A and the corresponding A of the passenger strand to a U. By changing the 5′ nucleotide, which is known to have no effect on target interactions of the siRNAs (50), the AGO2-selected vsiRNA can be transformed into an AGO1-specific vsiRNA, and the AGO1-selected vsiRNA can be transformed into an AGO2-specific vsiRNA (42,43).

We tested both sets of vsiRNAs with AGO1 and AGO2 in slicer assays with the TBSV A-region. Interestingly, when ‘adapted’ with their 5′ nucleotide to the applied AGO, we observed that each of the modified vsiRNAs were highly active, i.e. the 5′U-modified *esiRNA*209 was functional with AGO1, and the 5′A-modified *esiRNA*179 was functional with AGO2. However, when we applied ‘non-cognate’ 5′ nucleotide constellations of *esiRNA*179 with AGO1 or AGO2 and *esiRNA*209 with AGO1, we detected considerably lower slicing activities. The scenario was different with *esiRNA*209: in tests with AGO2, this RNA was very active, regardless of the 5′ nucleotide selected, which suggested a higher flexibility of AGO2 in the uptake of siRNAs with different 5′ termini (Figure 4E).

Taken together, we found that an siRNA is effective regardless of its AGO1- and AGO2-incorporation preference if the corresponding RISC cleavage site on the viral genome is accessible.

### *In vitro* identified *esiRNAs* efficiently protect plants against TBSV infections

**Figure 5.**
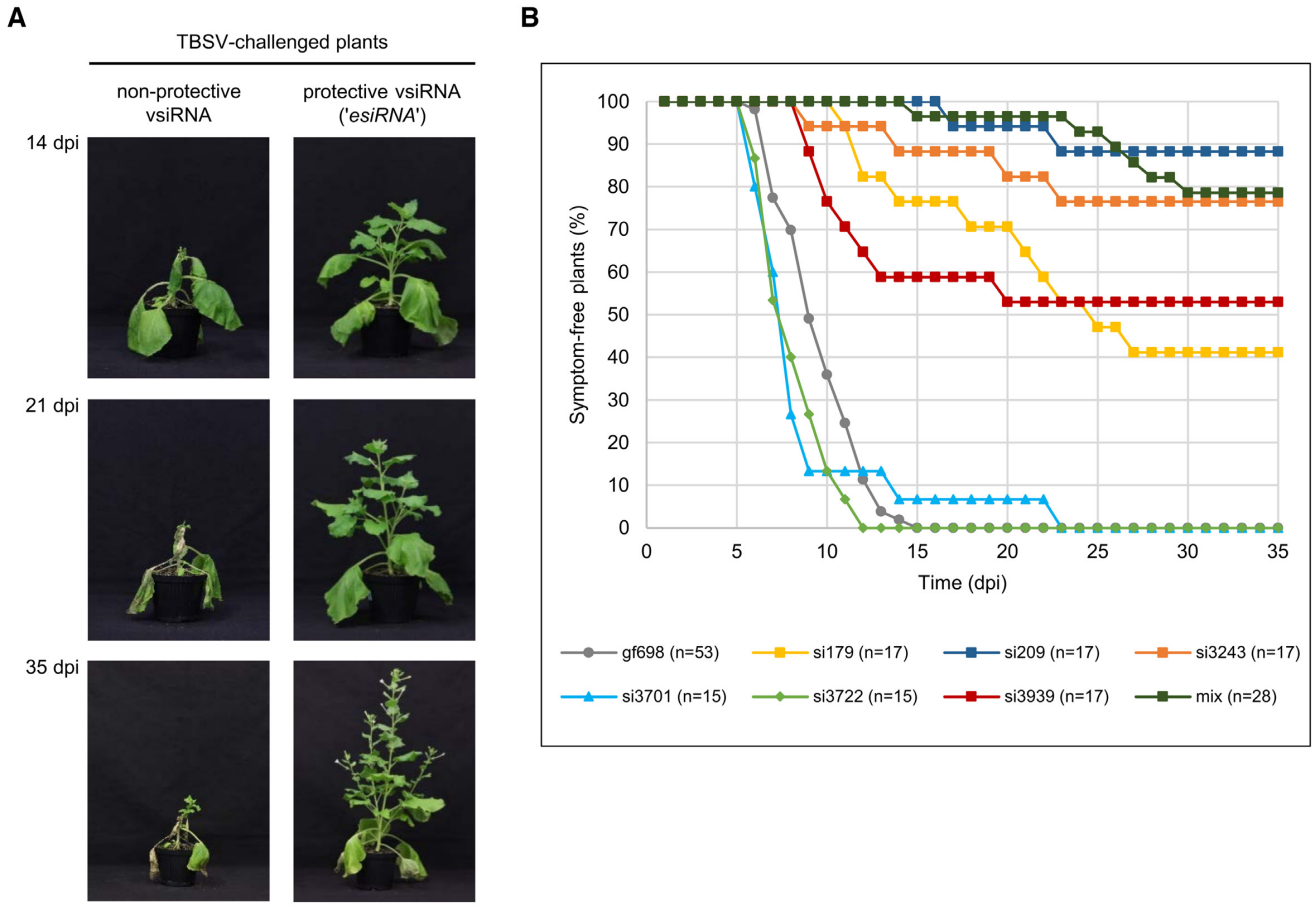
vsiRNAs with a high *in vitro* slicing activity (‘*esiRNAs*’) effectively protect plants against TBSV infections. (**A**) Comparison of *Nicotiana benthamiana* plants expressing a non-protective or a protective vsiRNA at different time points (days post infiltration, dpi) after TBSV challenge. TBSV derived vsiRNAs, including *esiRNAs*, were transiently produced as *MIR390*-based amiRNAs via agroinfiltration of plant leaves (35). Two days later, the same leaves were infiltrated with Agrobacteria containing a TBSV expression plasmid (see ‘Materials and Methods’ section). (**B**) Summary of the protective effect of individual vsiRNAs against TBSV infection. The siRNA gf698, targeting GFP mRNA was used as a negative control, i.e. equal amounts of Agrobacteria harboring an amiRNA construct expressing either a 5′U or a 5′A variant of this siRNA were mixed and infiltrated. In the case of the indicated siRNA mix, equal amounts of Agrobacteria harboring an amiRNA construct expressing each of the following vsiRNAs 179, 186, 207, 209, 221, 228 and 238 were mixed and inoculated. At least three independent ‘vaccination experiments’ were carried out in each case.

In the final series of experiments, we tested the protective potency of the identified *esiRNAs* in antiviral plant protection approaches. For this purpose, we applied an amiR-based system (35). We incorporated the sequences of *esiRNAs* or of control siRNAs listed below and shown in Table 2 into the *A. thaliana* microRNA390a backbone and transiently expressed these recombinant miRNAs in two leaves of 4-to 6-week-old *Nicotiana benthamiana* plants via*Agrobacterium tumefaciens*-based infiltration. Two days later, we ‘challenged’ the plants by infiltration of the same leaf-sites with *A. tumefaciens* containing a TBSV-expression plasmid. We grew the plants for an additional 35 days and monitored daily for symptom development such as wrinkled leaves or apical collapse. In the absence of protective measures, we observed that the applied TBSV ‘challenge’ led to 100% of the plants developing symptoms ranging from severe systemic necrosis to dying within this period, which indicated a massive viral infection (‘Materials and Methods’ section; Figure 5A).

**Table 2.** Summary of the plant vaccination/TBSV challenge experiments^a^

siRNA	5′ nt	*In vitro* cleavage of TBSV gRNA	Symptom-free plants	Symptom-free plants (%)	Symptom appearance (dpi)^b^
gf698	A+U	-	0/53	0	9.7±2.2
179	U	+	7/17	41	18.5±5.9
209	A	+++	15/17	88	20.0±4.2
3243	A	++	13/17	76	16.5±6.2
3701	A	-	0/15	0	8.9±4.4
3722	U	-	0/15	0	8.3±1.8
3939	U	+	9/17	53	11.8±3.6
mix^c^	A+U	N/A	22/28	79	25.0±5.3

^a^Each vsiRNA was evaluated in at least three independent experiments, vaccination with GFP-specific siRNA gf698 was included in every single experiment as a control. The efficiency of the *in vitro* cleavage is indicated: +++ best cleavage; ++ well detectable cleavage; + detectable cleavage, - no cleavage, N/A: not applicable (see also Figure 3 and Supplementary Figure S4).

^b^Mean ± SD.

^c^vsiRNAs 179, 186, 207, 209, 221, 228 and 238.

In a first approach, we tested the individual *esiRNAs* that we originally identified as AGO1-specific RNAs (*esiRNA*179 and *esiRNA*3939) and AGO2-specific RNAs (*esiRNA*209 and *esiRNA*3243) and that we observed to be the most effective in the *in vitro* slicer assays (Figure 3 and Supplementary Figure S4) for a protective effect. Second, we tested a combination of the seven vsiRNAs 179, 186, 207, 209, 221, 228 and 238 that were directed against the TBSV RNA 5′ terminus, which proved to be particularly accessible to AGO/RISC (Figure 4C). Third, we tested the two vsiRNAs 3722 (AGO1-specific) and 3701 (AGO2-specific) that were inactive in the *in vitro* slicer assay with the TBSV RNA (Figure 3), and we applied gf698 siRNAs with a 5′U or a 5′A as negative-controls.

Interestingly, each of the *esiRNAs* found to be highly active on the full-length TBSV genome *in vitro* yielded significant rates of plant protection. Actually, we found that the level of protection roughly correlated with the level of the *in vitro* slicing activity (Figure 5B and Table 2). That is, the vsiRNAs 3722 and 3701, which were not active in the *in vitro* slicer assay, remained non-protective. In contrast, *esiRNA*209 was most effective with a protection rate of ∼90%: while the control plants were 100% infected by TBSV and developed symptoms (see above), about 90% of the plants ‘vaccinated’ with *esiRNA209* remained unaffected. Correspondingly, *esiRNAs* 3243, 3939 and 179 yielded protection rates of 76%, 53% and 41%, respectively. The protection rate of the vsiRNA mixture was comparable to that of the individual *esiRNA*209 (Table 2 and ‘Discussion’ section). Another interesting observation was that the few plants that were not fully protected by the *esiRNAs* developed symptoms considerably later than the control plants (Figure 5B and Table 2).

It should be noted that protective ‘RNA vaccinations’ with *esiRNAs* were also obtained when the TBSV infection was carried out in a more natural way, namely by mechanical inoculation (‘rub in’) of the viral RNA (Supplementary Figure S5).

In sum, these findings suggest that the *esiRNAs*, which were identified with the described *in vitro* approach, indeed represent highly effective tools for antiviral plant protection.

## DISCUSSION

The fact that only a minimal number of siRNAs produced in the course of an RNA silencing immune response of the plant are effective against a pathogen is an important recent observation made and confirmed by various laboratories (9,16,22,23,51,52).

In view of the co-existence and co-evolution of plants and pathogens, it is not surprising that pathogenic RNA molecules such as viral RNAs have evolved in such a way that the RNA silencing reaction of the plant generates predominantly siRNAs that are not immunologically effective. The first attractive hypotheses about possible functions of such ‘non-effective’ siRNAs are currently being developed. For example, these siRNAs may function as decoys that saturate or mislead the silencing machinery and thus benefit the pathogen (53,54). Likewise, the pathogen-derived siRNAs may target cellular mRNAs that encode proteins involved in the plant’s immune response (22,55).

In contrast, we still have little understanding of the nature and properties of the immunologically effective *esiRNAs*, i.e. those vsiRNAs that actually act against the pathogen and are most interesting in terms of their potential application in crop protection approaches. A major reason for this is that we were yet unable to distinguish *esiRNAs* reliably and efficiently from the mass of silencing-generated non-effective vsiRNAs.

Several *in silico* approaches are available that predict potentially functional siRNAs, e.g. based on the identification of conserved elements and the prediction of theoretically accessible RNA structures of the target RNAs (56–59). However, such predicted siRNAs often prove ineffective *in planta*, as reliable predictions about RISC-accessible sites have not yet been possible (see below). Other methods identified functional siRNAs from mapping analyses of RISC-cleavage sites in viral RNAs isolated from infected plants (9,16,22,51). These procedures are technically difficult and unable to detect *esiRNAs* that are only present in low quantities. Furthermore, *esiRNAs* sequestered by viral suppressors of RNA silencing are not detected.

Here, we have developed an *in vitro*-based procedure that enables a systematic and simple identification as well as a functional characterization of *esiRNAs* of a given pathogen. Our approach offers several major advantages. The most obvious benefit is that the BYL-based assays can be reproducibly performed at medium throughput level *in vitro*. Thus, except for the final challenge experiments testing for plant protection, time-consuming infection studies with wt and/ or silencing-mutated (e.g. AGO-mutated) plants are replaced by a rapid assay procedure. The BYL imitates the plant’s silencing response rather effectively and besides reproducing the DCL-mediated RNA processing, the system enables specific screening with defined assembled AGO/RISC species, with pools or with single small RNAs and with target RNAs of choice. By limiting the screen to (-)vsiRNAs, we were able to further focus the *esiRNA* identification procedure on those candidates that target the silencing-accessible parental and/ or progeny viral (+)-strand RNA genomes (23,45).

The screen development process itself yielded several, valuable insights into the mechanisms of action of *esiRNAs*. First, we identified siRNAs that were only produced in small amounts by the DCLs and whose guide strands bind to the AGO proteins with high affinity. Several, but not all, of these siRNAs proved to be *esiRNAs*, confirming the assumption that the binding affinity of an siRNA duplex or individual siRNA strand to an AGO protein is a crucial but not exclusive determinant of the silencing capacity of this RNA. SiRNA/AGO interactions have already been known to depend critically on the size and the type of 5′ nucleotide. However, our data suggest that other, yet undefined siRNA features also affect AGO binding. This was most obvious with vsiRNA3516 (Table 1), which has the required size of 21 nt and a 5′A, but was selected with AGO1. Accordingly, an important aspect of future investigations will be to define these additional siRNA characteristics and to understand how these affect the plant’s immune response. Second, we found that the activity of an *esiRNA* also depends crucially on the ability of the corresponding RISC to access the target RNA. Similar observations were already reported by several labs that investigated the properties of RISC *in vitro* and *in vivo* (23,60–62). Considering the difficulty/impracticality of determining the structures of long RNA molecules *in silico* or *in vivo*, the possibility to apply the *in vitro* slicer assay for the detection of sites within a target RNAs that are accessible to RISC (Figures 3 and 4) proved to be a welcome asset.

One promising approach to attain antiviral resistance in the plant is to specifically stimulate RNA silencing (63). This can be achieved, for example, by transgenic expression or transient (topical) application of siRNAs, amiRNAs or dsRNAs (64–72). Current procedures aimed at inducing an antiviral RNA silencing reaction in plants are often based on the use of dsRNAs that include large parts of the viral RNAs. The limitation of these approaches is evident because in analogy to a normal infection, large vsiRNA pools are generated in the plant that contain only very few *esiRNAs*. Thus, to minimize decoy and off-target effects of ‘non-effective’ siRNAs, an essential goal in the development of highly effective plant protection methods based on silencing would, therefore, be the targeted and, potentially exclusive use of *esiRNAs* against a specific pathogen.

In fact, our combined *in vitro*/ *in vivo* approach yielded the proof-of-concept that *esiRNAs* identified in this way are highly protective *in planta*. Moreover, we found that *esiRNAs* operate in the context of both AGO1 and AGO2/RISC.

Effective RISC-mediated cleavage of the viral target RNA was found with *esiRNAs* that derived from different areas of the ORF-coding 3′-portion of the genome (*esiRNAs* 3243, 3939; Figures 3 and 5) as well as with a series of *e*siRNAs that derived from the same area within the TBSV RNA’s 5′-end (*esiRNAs* 179, 207, 209, 221, 228; Figure 4). According to an experimentally determined TBSV RNA secondary structure reported by Wu *et al.* (47; see also Figure 4A), the target regions of all these *esiRNAs* were actually considered weakly structured. However, the TBSV genome contains hundreds of such sites, which, in turn, confirmed the value of the *esiRNA* screening method used (see earlier ‘Discussion’ section). The particular accessibility of the TBSV 5′-end to RISC may also be explained by the involvement of this region in translation initiation. For example, it is conceivable that the long-range RNA–RNA interactions of a stem–loop structure close to the immediate TBSV RNA’s 5′-terminus with the CITE (cap-independent translational enhancer) in the 3′UTR that enables translation initiation and/or ribosome movement (73) expose nt 160–230 of the genome to such an extent that this region becomes susceptible to RISC activity.

Most importantly, we observed a significant correlation between the slicing activity of an siRNA *in vitro* and its protective competence. Obvious examples are the *esiRNAs* 209 and 3243. Both function in the same AGO protein, but with different efficacy, and the cleavage efficiency *in vitro* correlates directly with the protection competence of these siRNAs *in planta* (see Figures 3–5 and Supplementary Figure S4). This states that a high slicing activity of an siRNA on the cognate target RNA *in vitro* provides a surprisingly reliable indicator of the protective effect of this siRNA as an *esiRNA in planta*. Thus, screening for effective slicing of the target RNA of a pathogen *in vitro* turned out to be a remarkable informative criterion for the reliable identification of plant protective *esiRNAs*.

Here, we limited our analysis exclusively to 21 nt antivirally acting siRNAs, which are supposed to be mainly active in direct slicing of the viral RNA (15,16). However, in order to test whether the biogenesis of viral secondary siRNAs contributes significantly to the protection against TBSV, we have repeated some of the RNA ‘vaccination’ experiments with RDR6-silenced (RDR6i) *N. benthamiana* plants (74). RDR6 is a key player in the processes leading to the generation of secondary siRNAs (75,76). Interestingly, with the RDR6i plants, we observed essentially the same protection profile as with the wild-type plants (see Supplementary Figure S6 and Figure 5) suggesting that protection by the applied 21 nt *esiRNAs* does not require the RDR6-dependent production of secondary siRNAs. In view of these observations, we consider it next important to investigate 22 nt siRNAs in a similar way, as these siRNAs might trigger the production of RDR-dependent secondary siRNAs (77,78). In addition, 22 nt siRNAs are less efficiently captured by viral suppressors than 21 nt siRNAs (79).

Another interesting observation was that individual *esiRNAs* (e.g. *esiRNA*209) were almost completely protective and that the protective potential was not further increased by the use of an *esiRNA* mixture (Table 2). This may be simply because in the transiently administered mixture the individual *esiRNAs* were present in lower concentrations. Alternatively, this observation can be interpreted as indicating that 80–90% of protected plants already represent the maximum level of protection that can be achieved with the applied protection scheme.

A very obvious way to generate virus-resistant plants would be the transgenic expression of identified *esiRNAs*. However, the fact that transient applications of the RNAs are able to protect plants with the high efficiency found here increases the attractiveness of transient/ topical application approaches versus transgenic approaches. This is even more pertinent considering that transgenic plants that have been laboriously produced may show only limited protection against frequently mutating viruses. The transient *A. tumefaciens*-based amiR approaches used here are certainly unsuitable for the use of *esiRNAs* in practice (e.g. in greenhouses). A conceivable, alternative form of application is to generate and transiently apply short dsRNA molecules that consist almost exclusively of a few *esiRNA* sequences. Following topical application and uptake, which may be achieved in different ways (80), these dsRNAs are expected to be processed by the plant’s DCLs to produce the functional *esiRNAs*. Compared to conventional dsRNA-based methods, these RNAs should not only be considerably more effective but also minimize the risk of off-target effects (see above). However, future experiments will have to show whether 21 nt or perhaps 22 nt *esiRNAs* in this or other forms of application will also allow longer-lasting protection against viral pathogens than is possible in current transient approaches.

Importantly, with the established *esiRNA* identification screen, all types of RNA applications may now be quickly adapted to modified virus forms. In particular, we expect that the use of combinations of *esiRNAs* not only increases the speed and flexibility of transient treatments, but also significantly improves cross-protection against rapidly changing virus populations in transient as well as transgenic applications. Apart from the identification of *esiRNAs* that target viral RNAs, we anticipate this approach to be broadly applicable to a wide range of different functional RNAs. For example, it is conceivable that *esiRNAs* directed against mRNAs of nematodes, fungi or other pests can be identified in a similar way and used in plant protection measures.

## DATA AVAILABILITY

NGS datasets have been deposited in GEO under accession number GSE121644.

## Supplementary Material

gkz678_Supplemental_Files
