# Supplementary material for: Highly efficacious antiviral protection of plants by small interfering RNAs identified in vitro
Source: Nucleic Acids Res. 2019 Aug 21;47(17):9343–57. doi: 10.1093/nar/gkz678 (PMC6755098; doi:10.1093/nar/gkz678)
Supplement: gkz678_Supplemental_Files [file gkz678_supplemental_files.zip › Supplementary Figures_Gago_revised (1).docx]

**Supplementary Figures**

**
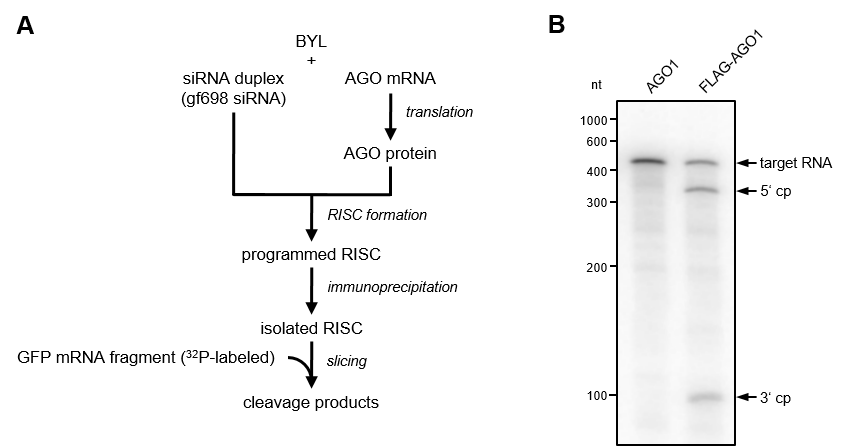
**

**Figure S1.** Active RISC isolated following *in vitro* assembly in BYL**.** (A) Schematic representation of the RISC immunoprecipitation and subsequent ‘slicer assay’. FLAG-tagged AGO1 was generated *via* *in vitro* translation in BYL in the presence of gf698 siRNA targeting the GFP mRNA. In a control reaction, the AGO1 protein was produced without FLAG-tag. The assembled RISCs were immunoprecipitated using an immobilized anti-FLAG antibody. Subsequently, a ^32^P-labeled GFP mRNA fragment was added and analyzed for siRNA-mediated cleavage. (B) Results of a ‘slicer assay’ with immunoprecipitated RISC that was generated in the presence of gf698 siRNA. Cleavage products (cp) are indicated.

**
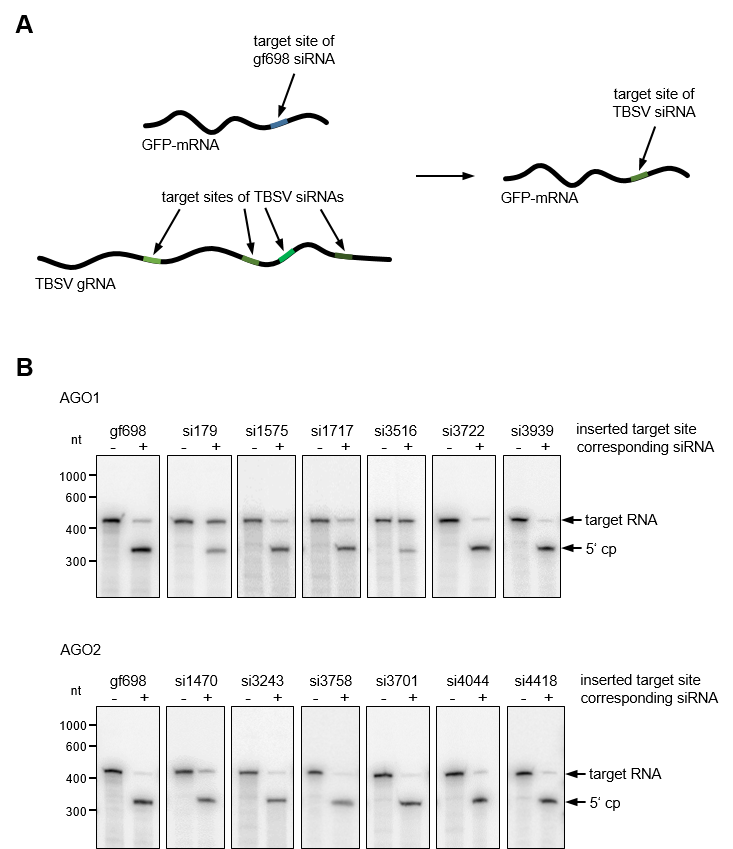
**

**Figure S2.** Inaccessibility of target sites to RISC explains the lack of activity of various siRNAs on the TBSV genome. (A) The target sequences of the TBSV siRNAs that were earlier identified and tested for cleavage of the genomic TBSV RNA (see Figure 3) were individually inserted into the context of the GFP mRNA, thereby replacing the target site of the gf698 siRNA. (B) ‘Slicer assays’ with these TBSV siRNAs and the modified GFP mRNA substrates containing the corresponding target site. The assay was essentially carried out as described in Figure 3. The 5’ cleavage products (cp) are indicated.

**
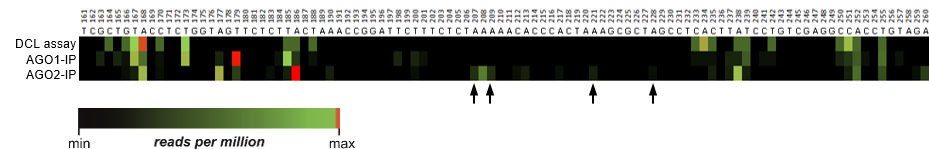
**

**Figure S3.** Heat map of 21 nt TBSV (-)siRNAs mapping to the 5’ part of the TBSV genome. Numbers indicate the nucleotide position of the TBSV genomic RNA. Arrows highlight the 5’-nucleotide positions of candidate (-)vsiRNAs that explained the cleavage products observed in the slicer assay with AGO2 and the ds TBSV fragment A (Figure 4).


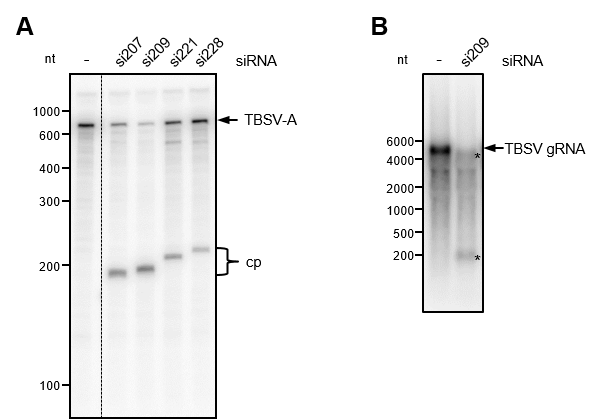


**Figure S4.** vsiRNA209 mediates highly efficient slicing of TBSV RNAs. (A) ‘Slicer assay’ with different TBSV siRNAs. The assay was carried out with AGO2 and TBSV RNA fragment A as described in Figure 3 and 4. However, in comparison with Fig. 3 and 4, reduced amounts (10%) of the indicated vsiRNAs and a tenfold excess of gf698 competitor siRNA were used. Cleavage products (cp) are indicated. (B) vsiRNA209 mediates efficient cleavage of full-length TBSV genomic RNA. The assay was carried out with AGO2 essentially as described in Figure 3. Cleavage products are indicated by asterisks.

**Figure S5.** The TBSV-derived *esiRNAs* 179 and 209 effectively protect *N. benthamiana* plants against TBSV infection, which is triggered by mechanically inoculated viral RNA. The *esiRNAs* and the control siRNA gf698, targeting GFP mRNA, were transiently produced as *MIR390*-based amiRNAs via agroinfiltration of plant leaves. Two days later, *in vitro* transcribed TBSV genomic RNA was rubbed onto the same leaves using a total amount of 10 ng RNA/plant (see Material and Methods). Two independent ‘vaccination experiments’ were carried out.

**Figure S6.** TBSV-derived *esiRNAs* effectively protect RDR6i *N. benthamiana* plants against TBSV infection. Tested were *esiRNAs* 179 and 209, and, as controls, the non-protective siRNAs 3701 and 3722 and siRNA gf698, which targets GFP mRNA (see Table 2 and Figure 5). The siRNAs were transiently produced as *MIR390*-based amiRNAs via agroinfiltration of plant leaves. Two days later, the same leaves were infiltrated with Agrobacteria containing a TBSV expression plasmid (see Material and Methods). Two independent ‘vaccination experiments’ were carried out.
