# Supplementary material for: Highly efficacious antiviral protection of plants by small interfering RNAs identified in vitro
Source: Nucleic Acids Res. 2019 Aug 21;47(17):9343–57. doi: 10.1093/nar/gkz678 (PMC6755098; doi:10.1093/nar/gkz678)
Supplement: gkz678_Supplemental_Files [file gkz678_supplemental_files.zip › Supplementary Tables_NAR.pdf]

Supplementary Table S1. DNA oligonucleotides used in this study

oligonucleotide	sequence	purpose
agf698_21U_1f	TGTATAGTTCATCCATGCCATGTGTATGATGATCACATTCGTTATCTATTTTTTACACATGGCAGGGATGAACTA	amiRNA cloning in binary vector pMDC32B-AtMIR390a-B/c
agf698_21U_1r	AATGTAGTTCATCCCTGCCATGTGTAAAAAATAGATAACGAATGTGATCATCATACACATGGCATGGATGAACTA	amiRNA cloning in binary vector pMDC32B-AtMIR390a-B/c
agf698_21A_1f	TGTAAAGTTCATCCATGCCATGTGTATGATGATCACATTCGTTATCTATTTTTTACACATGGCAGGGATGAACTT	amiRNA cloning in binary vector pMDC32B-AtMIR390a-B/c
agf698_21A_1r	AATGAAGTTCATCCCTGCCATGTGTAAAAAATAGATAACGAATGTGATCATCATACACATGGCATGGATGAACTT	amiRNA cloning in binary vector pMDC32B-AtMIR390a-B/c
asiRNA179m_1f	TGTATGATGGTCTCCATGTCGCTTGATGATGATCACATTCGTTATCTATTTTTTCAAGCGACATTGAGACCATCA	amiRNA cloning in binary vector pMDC32B-AtMIR390a-B/c
asiRNA179m_1r	AATGTGATGGTCTCAATGTCGCTTGAAAAAATAGATAACGAATGTGATCATCATCAAGCGACATGGAGACCATCA	amiRNA cloning in binary vector pMDC32B-AtMIR390a-B/c
asiRNA186m_1f	TGTAATTCTCTTGATGGTCTCCATGATGATGATCACATTCGTTATCTATTTTTTCATGGAGACCCTCAAGAGAAT	amiRNA cloning in binary vector pMDC32B-AtMIR390a-B/c
asiRNA186m_1r	AATGATTCTCTTGAGGGTCTCCATGAAAAAATAGATAACGAATGTGATCATCATCATGGAGACCATCAAGAGAAT	amiRNA cloning in binary vector pMDC32B-AtMIR390a-B/c
asiRNA207m_1f	TGTAATCTCTTTCTTAGGCCAAATCATGATGATCACATTCGTTATCTATTTTTTGATTTGGCCTCAGAAAGAGAT	amiRNA cloning in binary vector pMDC32B-AtMIR390a-B/c
asiRNA207m_1r	AATGATCTCTTTCTGAGGCCAAATCAAAAAATAGATAACGAATGTGATCATCATGATTTGGCCTAAGAAAGAGAT	amiRNA cloning in binary vector pMDC32B-AtMIR390a-B/c
asiRNA209m_1f	TGTAAAATCTCTTTCTTAGGCCAAAATGATGATCACATTCGTTATCTATTTTTTTTTGGCCTAATAAAGAGATTT	amiRNA cloning in binary vector pMDC32B-AtMIR390a-B/c
asiRNA209m_1r	AATGAAATCTCTTTATTAGGCCAAAAAAAAATAGATAACGAATGTGATCATCATTTTGGCCTAAGAAAGAGATTT	amiRNA cloning in binary vector pMDC32B-AtMIR390a-B/c
asiRNA221m_1f	TGTAAATCACCCACAAAAATCTCTTATGATGATCACATTCGTTATCTATTTTTTAAGAGATTTTGGTGGGTGATT	amiRNA cloning in binary vector pMDC32B-AtMIR390a-B/c
asiRNA221m_1r	AATGAATCACCCACCAAAATCTCTTAAAAAATAGATAACGAATGTGATCATCATAAGAGATTTTTGTGGGTGATT	amiRNA cloning in binary vector pMDC32B-AtMIR390a-B/c
asiRNA228m_1f	TGTAATCGCGAAATCACCCACAAAAATGATGATCACATTCGTTATCTATTTTTTTTTTTGTGGGTTATTTCGCGAT	amiRNA cloning in binary vector pMDC32B-AtMIR390a-B/c
asiRNA228m_1r	AATGATCGCGAAATAACCCACAAAAAAAAAATAGATAACGAATGTGATCATCATTTTTGTGGGTGATTTCGCGAT	amiRNA cloning in binary vector pMDC32B-AtMIR390a-B/c
asiRNA238m_1f	TGTAATTCACTCCGATCGCGAAATCATGATGATCACATTCGTTATCTATTTTTTGATTTCGCGAGCGGAGTGAAT	amiRNA cloning in binary vector pMDC32B-AtMIR390a-B/c
asiRNA238m_1r	AATGATTCACTCCGCTCGCGAAATCAAAAAATAGATAACGAATGTGATCATCATGATTTCGCGATCGGAGTGAAT	amiRNA cloning in binary vector pMDC32B-AtMIR390a-B/c
asiRNA3243m_1f	TGTAATTCGCCAACTCAACTCTATCATGATGATCACATTCGTTATCTATTTTTTGATAGAGTTGCGTTGGCGAAT	amiRNA cloning in binary vector pMDC32B-AtMIR390a-B/c
asiRNA3243m_1r	AATGATTCGCCAACGCAACTCTATCAAAAAATAGATAACGAATGTGATCATCATGATAGAGTTGAGTTGGCGAAT	amiRNA cloning in binary vector pMDC32B-AtMIR390a-B/c
asiRNA3701m_1f	TGTAAAAAACGCACTGTCTGTACCTATGATGATCACATTCGTTATCTATTTTTTAGGTACAGACCGTGCGTTTTT	amiRNA cloning in binary vector pMDC32B-AtMIR390a-B/c
asiRNA3701m_1r	AATGAAAAACGCACGGTCTGTACCTAAAAAATAGATAACGAATGTGATCATCATAGGTACAGACAGTGCGTTTTT	amiRNA cloning in binary vector pMDC32B-AtMIR390a-B/c
asiRNA3722m_1f	TGTATTAGAGACAGTACAATTTATGATGATGATCACATTCGTTATCTATTTTTTCATAAATTGTCCTGTCTCTAA	amiRNA cloning in binary vector pMDC32B-AtMIR390a-B/c
asiRNA3722m_1r	AATGTTAGAGACAGGACAATTTATGAAAAAATAGATAACGAATGTGATCATCATCATAAATTGTACTGTCTCTAA	amiRNA cloning in binary vector pMDC32B-AtMIR390a-B/c
asiRNA3939m_1f	TGTATTCACTGTTAGCTTGTTCCCTATGATGATCACATTCGTTATCTATTTTTTAGGGAACAAGATAACAGTGAA	amiRNA cloning in binary vector pMDC32B-AtMIR390a-B/c
asiRNA3939m_1r	AATGTTCACTGTTATCTTGTTCCCTAAAAAATAGATAACGAATGTGATCATCATAGGGAACAAGCTAACAGTGAA	amiRNA cloning in binary vector pMDC32B-AtMIR390a-B/c
T7pro2G	CCCTAATACGACTCACTATAGG	generation of template DNA for *in vitro* transcription of TBSV fragment A, (+)-strand
TVGM_JS1r	CCTTAAGTTCCCTTGCCGGC	generation of template DNA for *in vitro* transcription of TBSV fragment A, (+)-strand
T7TVGM_JS2f	TGTAATACGACTCACTATAGGAGCCGGCAAGGGAACTTAAG	generation of template DNA for *in vitro* transcription of TBSV fragment B, (+)-strand
TVGM_JS2r	CATTGTACCTCGGGTTTCGAG	generation of template DNA for *in vitro* transcription of TBSV fragment B, (+)-strand
T7TVGM_JS3f	TGTAATACGACTCACTATAGGCAGCACCTCGGGTGATTCAG	generation of template DNA for *in vitro* transcription of TBSV fragment C, (+)-strand
TVGM_JS3r	GTAATTGCTTTGTACTCACCGG	generation of template DNA for *in vitro* transcription of TBSV fragment C, (+)-strand
T7TVGM_JS4f	TGTAATACGACTCACTATAGGCACACACGCAGGATAGACAC	generation of template DNA for *in vitro* transcription of TBSV fragment D, (+)-strand
TVGM_JS4r	CGCAGGACACACTAAATTAGAG	generation of template DNA for *in vitro* transcription of TBSV fragment D, (+)-strand
T7TVGM_JS5f	TGTAATACGACTCACTATAGGTATCACATCTGCCACAGTAC	generation of template DNA for *in vitro* transcription of TBSV fragment E, (+)-strand
TVDIWT1r	GGGCTGCATTTCTGCAATGTTC	generation of template DNA for *in vitro* transcription of TBSV fragment E, (+)-strand
TVmTra1f	GGAAATTCTCCAGGATTTCTCGAC	generation of template DNA for *in vitro* transcription of TBSV fragment A, (-)-strand
T7TVGM1r	TGTAATACGACTCACTATAGGCCTTAAGTTCCCTTGCCGGC	generation of template DNA for *in vitro* transcription of TBSV fragment A, (-)-strand
TraTVGM_JS2f	GGAGCCGGCAAGGGAACTTAAG	generation of template DNA for *in vitro* transcription of TBSV fragment B, (-)-strand
T7TVGM_JS2r	CCCTAATACGACTCACTATAGGCATTGTACCTCGGGTTTCGAG	generation of template DNA for *in vitro* transcription of TBSV fragment B, (-)-strand
TraTVGM_JS3f	GGCAGCACCTCGGGTGATTCAG	generation of template DNA for *in vitro* transcription of TBSV fragment C, (-)-strand
T7TVGM_JS3r	CCCTAATACGACTCACTATAGGTAATTGCTTTGTACTCACCGG	generation of template DNA for *in vitro* transcription of TBSV fragment C, (-)-strand
TraTVGM_JS4f	GGCACACACGCAGGATAGACAC	generation of template DNA for *in vitro* transcription of TBSV fragment D, (-)-strand
T7TVGM_JS4r	CCCTAATACGACTCACTATAGGCGCAGGACACACTAAATTAGAG	generation of template DNA for *in vitro* transcription of TBSV fragment D, (-)-strand
TraTVGM_JS5f	GGTATCACATCTGCCACAGTAC	generation of template DNA for *in vitro* transcription of TBSV fragment E, (-)-strand
T7TVmTra1r	CCCTAATACGACTCACTATAGGGCTGCATTTCTGCAATGTTCC	generation of template DNA for *in vitro* transcription of TBSV fragment E, (-)-strand
pGFP-C1mut1f	GGAACTACAAGACACGTGCTG	replacement of the target site of siRNA gf698 in plasmid pGFP-C1 by two *Bpi* I sites
pGFP-C1mut1r	CATGCCATGGttGAAGACtcATCCCAGCAGCTGTTACAAAC	replacement of the target site of siRNA gf698 in plasmid pGFP-C1 by two *Bpi* I sites
pGFP-C1mut2f	CATGCCATGGttGAAGACtcTACAAGTCCGGACTCAGATCTC	replacement of the target site of siRNA gf698 in plasmid pGFP-C1 by two *Bpi* I sites
pGFP-C1mut2r	TTGCCGATTTCGGCCTATTGG	replacement of the target site of siRNA gf698 in plasmid pGFP-C1 by two *Bpi* I sites
tsTVsi179m1f	GGATACAAGCGACATGGAGACCATCA	insertion of the target site of a TBSV siRNA in plasmid pGFP-C1
tsTVsi179m1r	TGTATGATGGTCTCCATGTCGCTTGT	insertion of the target site of a TBSV siRNA in plasmid pGFP-C1
tsTVsi1470m1f	GGATGAGCCGTGGAGAGTCTGCATAT	insertion of the target site of a TBSV siRNA in plasmid pGFP-C1
tsTVsi1470m1r	TGTAATATGCAGACTCTCCACGGCTC	insertion of the target site of a TBSV siRNA in plasmid pGFP-C1
tsTVsi1575m1f	GGATCTCGGGTGATTCAGCCTCGAAA	insertion of the target site of a TBSV siRNA in plasmid pGFP-C1
tsTVsi1575m1r	TGTATTTCGAGGCTGAATCACCCGAG	insertion of the target site of a TBSV siRNA in plasmid pGFP-C1
tsTVsi1717m1f	GGATAGGTGCAATTTTCCGGGCTAAA	insertion of the target site of a TBSV siRNA in plasmid pGFP-C1
tsTVsi1717m1r	TGTATTTAGCCCGGAAAATTGCACCT	insertion of the target site of a TBSV siRNA in plasmid pGFP-C1
tsTVsi3243m1f	GGATTGATAGAGTTGAGTTGGCGAAT	insertion of the target site of a TBSV siRNA in plasmid pGFP-C1
tsTVsi3243m1r	TGTAATTCGCCAACTCAACTCTATCA	insertion of the target site of a TBSV siRNA in plasmid pGFP-C1
tsTVsi3516m1f	GGATTGGCGCTCTTGTCACAGCATCT	insertion of the target site of a TBSV siRNA in plasmid pGFP-C1
tsTVsi3516m1r	TGTAAGATGCTGTGACAAGAGCGCCA	insertion of the target site of a TBSV siRNA in plasmid pGFP-C1
tsTVsi3701m1f	GGATTAGGTACAGACAGTGCGTTTTT	insertion of the target site of a TBSV siRNA in plasmid pGFP-C1
tsTVsi3701m1r	TGTAAAAAACGCACTGTCTGTACCTA	insertion of the target site of a TBSV siRNA in plasmid pGFP-C1
tsTVsi3722m1f	GGATTCATAAATTGTACTGTCTCTAA	insertion of the target site of a TBSV siRNA in plasmid pGFP-C1
tsTVsi3722m1r	TGTATTAGAGACAGTACAATTTATGA	insertion of the target site of a TBSV siRNA in plasmid pGFP-C1
tsTVsi3758m1f	GGATTGACATTCACATCTACCGGTAT	insertion of the target site of a TBSV siRNA in plasmid pGFP-C1
tsTVsi3758m1r	TGTAATACCGGTAGATGTGAATGTCA	insertion of the target site of a TBSV siRNA in plasmid pGFP-C1
tsTVsi3939m1f	GGATTAGGGAACAAGCTAACAGTGAA	insertion of the target site of a TBSV siRNA in plasmid pGFP-C1
tsTVsi3939m1r	TGTATTCACTGTTAGCTTGTTCCCTA	insertion of the target site of a TBSV siRNA in plasmid pGFP-C1
tsTVsi4044m1f	GGATTAACGATGAGACGAATTCGAAT	insertion of the target site of a TBSV siRNA in plasmid pGFP-C1
tsTVsi4044m1r	TGTAATTCGAATTCGTCTCATCGTTA	insertion of the target site of a TBSV siRNA in plasmid pGFP-C1
tsTVsi4418m1f	GGATAAGGCGAGTAAGACAGACTCTT	insertion of the target site of a TBSV siRNA in plasmid pGFP-C1
tsTVsi4418m1r	TGTAAAGAGTCTGTCTTACTCGCCTT	insertion of the target site of a TBSV siRNA in plasmid pGFP-C1
HDVrib1f	GGGTCGGCATGGCATCTCCAC	fusion of a HDV ribozyme-encoding sequence to TBSV cDNA
HDVrib3r	CGGAATTCTCTAGACTTCTCCCTTAGCCTACCGAAG	fusion of a HDV ribozyme-encoding sequence to TBSV cDNA
TBSV_BGW_1f	TTTGGTCTCGATCCGGAAATTCTCCAGGATTTCTCG	cloning of TBSV cDNA including a HDV ribozyme-encoding sequence in binary vector pVM-BGW
TBSV_BGWRib_2r	TTTGGTCTCTAAGCCGGAATTCTCTAGACTTCTCCC	cloning of TBSV cDNA including a HDV ribozyme-encoding sequence in binary vector pVM-BGW
BGWmCherry2f	GACCATGAAGACCGATCCATGGTGAGCAAGGGCGAGG	cloning of mCherry ORF in binary vector
BGWmCherry2r	GACCATGAAGACCGAAGCTTACTTGTACAGCTCGTCCATG	cloning of mCherry ORF in binary vector

Supplementary Table S2. RNA oligonucleotides used in this study

oligonucleotide	sequence	purpose
gf698-21U gs	uaguucauccaugccaugugu	guide strand of gf698 siRNA, 5'U
gf698-21U ps	acauggcauggaugaacuaua	passenger strand of gf698 siRNA
gf698-21A gs	aaguucauccaugccaugugu	guide strand of gf698 siRNA, 5'A
gf698-21A ps	acauggcauggaugaacuuua	passenger strand of gf698 siRNA
si179 gs	ugauggucuccaugucgcuug	guide strand of TBSV siRNA 179
si179 ps	agcgacauggagaccaucaag	passenger strand of TBSV siRNA 179
si186 gs	auucucuugauggucuccaug	guide strand of TBSV siRNA 186
si186 ps	uggagaccaucaagagaauga	passenger strand of TBSV siRNA 186
si207 gs	aucucuuucuuaggccaaauc	guide strand of TBSV siRNA 207
si207 ps	uuuggccuaagaaagagauuu	passenger strand of TBSV siRNA 207
si209 gs	aaaucucuuucuuaggccaaa	guide strand of TBSV siRNA 209
si209 ps	uggccuaagaaagagauuuuu	passenger strand of TBSV siRNA 209
si221 gs	aaucacccacaaaaaucucuu	guide strand of TBSV siRNA 221
si221 ps	gagauuuuugugggugauuuc	passenger strand of TBSV siRNA 221
si228 gs	aucgcgaaaucacccacaaaa	guide strand of TBSV siRNA 228
si228 ps	uugugggugauuucgcgaucg	passenger strand of TBSV siRNA 228
si238 gs	auucacuccgaucgcgaaauc	guide strand of TBSV siRNA 238
si238 ps	uuucgcgaucggagugaauag	passenger strand of TBSV siRNA 238
si1470 gs	auaugcagacucuccacggcu	guide strand of TBSV siRNA 1470
si1470 gs	ccguggagagucugcauauca	passenger strand of TBSV siRNA 1470
si1575 gs	uuucgaggcugaaucacccga	guide strand of TBSV siRNA 1575
si1575 ps	gggugauucagccucgaaacc	passenger strand of TBSV siRNA 1575
si1717 gs	uuuagcccggaaaauugcacc	guide strand of TBSV siRNA 1717
si1717 ps	ugcaauuuuccgggcuaaaug	passenger strand of TBSV siRNA 1717
si3243 gs	auucgccaacucaacucuauc	guide strand of TBSV siRNA 3243
si3243 ps	uagaguugaguuggcgaauua	passenger strand of TBSV siRNA 3243
si3516 gs	agaugcugugacaagagcgcc	guide strand of TBSV siRNA 3516
si3516 ps	cgcucuugucacagcaucugg	passenger strand of TBSV siRNA 3516
si3701 gs	aaaaacgcacugucuguaccu	guide strand of TBSV siRNA 3701
si3701 ps	guacagacagugcguuuuuca	passenger strand of TBSV siRNA 3701
si3722 gs	uuagagacaguacaauuuaug	guide strand of TBSV siRNA 3722
si3722 ps	uaaauuguacugucucuaacc	passenger strand of TBSV siRNA 3722
si3758 gs	auaccgguagaugugaauguc	guide strand of TBSV siRNA 3758
si3758 ps	cauucacaucuaccgguauca	passenger strand of TBSV siRNA 3758
si3939 gs	uucacuguuagcuuguucccu	guide strand of TBSV siRNA 3939
si3939 ps	ggaacaagcuaacagugaacg	passenger strand of TBSV siRNA 3939
si4044 gs	auucgaauucgucucaucguu	guide strand of TBSV siRNA 4044
si4044 ps	cgaugagacgaauucgaauca	passenger strand of TBSV siRNA 4044
si4418 gs	aagagucugucuuacucgccu	guide strand of TBSV siRNA 4418
si4418 ps	gcgaguaagacagacucuuca	passenger strand of TBSV siRNA 4418
